# Comparative Investigation of Thermal and Structural Behavior in Renewably Sourced Composite Films of Even-Even Nylons (610 and 1010) with Silk Fibroin

**DOI:** 10.3390/polym10091029

**Published:** 2018-09-15

**Authors:** Kayla A. Callaway, Ye Xue, Vincent Altimari, Guoxiang Jiang, Xiao Hu

**Affiliations:** 1 Department of Physics & Astronomy, Rowan University, Glassboro, NJ 08028, USA; callaw44@students.rowan.edu (K.A.C.); altimariv0@students.rowan.edu (V.A.); jiangg0@students.rowan.edu (G.J.); 2 Department of Biomedical Engineering, Rowan University, Glassboro, NJ 08028, USA; xuey5@rowan.edu; 3 Department of Molecular and Cellular Biosciences, Rowan University, Glassboro, NJ 08028, USA

**Keywords:** silk, nylon, miscibility, composite biomaterial, phase, crystal

## Abstract

As the average life expectancy continues to increase, so does the need for resorbable materials designed to treat, augment, or replace components and functions of the body. Naturally occurring biopolymers such as silks are already attractive candidates due to natural abundance and high biocompatibility accompanied by physical properties which are easily modulated through blending with another polymer. In this paper, the authors report on the fabrication of biocomposite materials made from binary blends of *Bombyx mori* silk fibroin (SF) protein and renewably sourced low molecular weight nylon 610 and high molecular weight nylon 1010. Films were characterized using scanning electron microscopy (SEM), Fourier-transform infrared (FTIR) spectroscopy, differential scanning calorimetry (DSC) and thermogravimetric analysis (TGA). Results of this study demonstrated that enhanced structural and thermal properties were achievable in composite films SF-N610/N1010 due to their chemical similarity and the possible formation of hydrogen bonds between nylon and silk molecular chains. This study provides useful insight into the sustainable design of functional composite materials for biomedical and green technologies.

## 1. Introduction

Highly renowned for its impressive strength and luster, silk has served as an important and versatile commodity material for centuries. Beyond its traditional and widespread use in textiles, silk's abundance of desirable properties offers great potential for use in a range of advanced materials applications. In particular, silk’s remarkable mechanical strength, flexibility, low immunogenicity, high oxygen permeability, and overall good biocompatibility [1,2,3,4] make it an excellent biomaterial [5]. These attributes complemented by its material versatility have promoted its fabrication into different functional material forms including hydrogels [6], nanoparticles [7], fibers [8], and a variety of composite materials [9,10,11]. It has already been demonstrated that regenerated silk-based materials serve great utility in a range of cutting-edge biomedical applications, including cell adhesion and growth [12], tissue engineering and regeneration [13,14], and drug delivery [2,7].

Derived from *Bombyx mori* cocoons, silk fibroin (SF) is a fibrous biopolymer consisting of repeating glycine-alanine/serine dipeptides responsible for beta-sheet crystal structures mixed with amorphous regions rich in large amino acids [3,15]. These repeating amino acid sequences and their interactions are what govern many of the properties observed in silk-based materials including mechanical and thermal behavior as well as hydrophobicity and biocompatibility [6,13]. An understanding of these structure–property relationships is crucial for good materials design. Although it offers desirable properties such as superior toughness, regenerated SF does have certain drawbacks such as brittleness [4]. Nevertheless, silk's greatest advantage over other materials is perhaps the ease with which its properties can be tuned or modified [9,16,17,18]. While various solvent treatments [19,20] and other processing techniques have been effective in preserving or improving silk properties, another approach is to blend it with another material such as cellulose [9], gelatin [21], polyvinyl alcohol (PVA) [22], and others. However, blending SF with another polymer can very often lead to severe phase separation [23]. When designing silk-based composite materials with the goal of achieving certain properties, researchers must consider miscibility as an important requirement in the selection of suitable blends [24].

Nylon is a family of semi-crystalline synthetic polymers made up of polyethylene segments separated by peptide units which can be arranged in either a parallel or antiparallel structure. Similar to silk, the conformation of nylon’s peptides results in the unique hydrogen bonding responsible for many of its attractive properties [25]. Originally intended to serve as a synthetic substitute to materials such as silk, nylon quickly evolved into a preferred material endowed with useful properties of its own, including good thermal stability, toughness, stiffness, and resistance to fatigue and abrasion [26]. The popularity of nylon as a versatile thermoplastic is largely due to its facile synthesis and the ability to easily tailor its attributes for different applications. Properties like moisture absorption, moduli, melting point, low-temperature impact strength, and resistance to metal salts and acids can be modified by changing the amide density [25]. Similar to silk, such properties may also be altered through post-synthesis treatments [27] or blending. 

Nylon and silk have great potential as candidates to modify each other. Chemical similarity between the polymers suggests possible miscibility. However, further motivation stems from the possibility that some interaction between molecular chains of silk and nylon will promote an altered structural conformation, leading to a difference in crystallization behavior and properties. Several types of nylons exist and are classified by their structural geometries. Of particular interest for this study, though, are nylons of the even-even type. Even-even nylons are distinguished by centers of symmetry along the molecule and have no sense of direction [28]. For this type of nylon, the alpha phase is the stable structure characterized by stacked planar sheets of hydrogen-bonded molecules existing as a tricyclic, fully extended zigzag conformation [28]. While no distinct beta phase is observed in even-even nylons, they do assume a metastable gamma-phase structure comprised of pleated sheets of methylene units exhibiting hydrogen-bonding between sheets instead of within sheets as is observed in the alpha phase. Under certain conditions, the gamma form can be converted to the alpha form [25].

SF has already been blended with different nylons to create a variety of materials with enhanced properties, supporting the conclusion that this combination is both functional and versatile. Cebe and colleagues [29] reported on enhanced thermal stability of SF-Nylon6 films ascribed to the strong interaction between the homopolymer, likely the result of hydrogen bonding between the silk and nylon side chains. Liu et al. [30] found that SF and Nylon 66 can be combined to form films with good miscibility at most composition ratios. Excluding the 50 wt % SF blend, they reported strong interaction leading to the co-crystallization of SF and N66, noting that a small amount of SF is likely to crystallize with Nylon 66, while the majority of SF may interact with N66 to form an amorphous composite.

While several researchers have investigated the modified properties of some silk-nylon composite materials, there has been a lack of attention paid to the effects of different amide densities in nylon on the overall composite properties. In this paper, the authors report on the fabrication and comparative investigation of the structural and thermal behavior in binary composite films fabricated from one of two forms of even-even nylons differing in molecular weight (N610 and N1010) with *Bombyx mori* silk at various composition ratios by solution cast method. Film synthesis utilized a CaCl_2_–formic acid solvent to cast films followed by the removal of Ca^+^ ions to allow for increased beta-sheet crystallinity. Films were characterized through morphological, structural, and thermal analysis. FTIR results confirmed that post-cast removal of Ca^+^ ions induced beta-sheet confirmation in SF. Further investigation using thermal analysis revealed that an increase in silk concentration led to a decrease in metastable gamma-phase nylon crystals and an increase of stable alpha-phase crystals for nylon 610 and nylon 1010. Improved thermal stability of silk was also observed when blended with nylon. 

## 2. Materials and Methods

### 2.1. Raw Materials

Zytel^®^ Renewably Sourced Polyamide nylons PA610 and PA1010 were received in pellets from Dupont^TM^ (Wilmington, DE, USA) as a gift (Dr. Bryan Sauer) and were not further purified. Cocoons of *Bombyx mori* silk were obtained from Treenway Silks (Lakewood, CO, USA). The cocoons were degummed to remove the sericin coating and extract the fibers by boiling in a 0.02 M NaHCO_3_ (Sigma-Aldrich, St. Louis, MO, USA) solution for 15 min followed by thorough rinsing with deionized water. The degummed silk fibers were air dried overnight, and put into a vacuum oven at room temperature for one day to remove surface moisture [31]. ACS Grade 98% Formic Acid was purchased from EMD Millipore Corporation (Burlington, MA, USA), ACS Grade Calcium Chloride Anhydrous was purchased from AMRESCO Inc. (Solon, OH, USA), and both were used as purchased.

### 2.2. Material Synthesis

Separate lines of polyamide composite films were created using nylon 610 and nylon 1010 blended with *Bombyx mori* silk at various composition ratios by solution cast method. Solutions of formic acid and CaCl_2_ (30 mg/mL) dissolved SF and nylon 610 separately at 15% *w*/*v* as well as SF and nylon 1010 separately at 10% *w*/*v*. Weight compositions of nylon solutions were determined by their respective solubility in formic acid and conditions for successful formation of casted films which are dependent on their molecular weights.

Bulk solutions of the nylons in formic acid were dissolved at approximately 55 °C and stirred at 1000 rpm for 2 h. To avoid degradation from extended exposure to formic acid, the rapid dissolution of silk at room temperature began only when the nylons were almost entirely dissolved. Silk was dissolved in increments to prevent the fibers from aggregating and forming a gel. Silk and nylon solutions were centrifuged to remove impurities and then combined at room temperature at the corresponding volumetric ratios to make seven different 5 mL samples for each type of nylon. Once combined, the samples were vortexed for 5 min and immediately cast on polydimethylsiloxane (PDMS) substrates. Films were left to dry overnight in the fume hood. The following day, films were soaked in water for 30 min to remove CaCl_2_ and then thoroughly dried via vacuum. For the remainder of the study, the samples are referred to in the following format: LN for Nylon 610 (low molecular weight nylon) and HN for Nylon 1010 (high molecular weight nylon). The number which follows (S%) is the percentage of silk in the film and is specified in each figure. For example, LNS30% is a composite film with 30 wt % silk and 70 wt % nylon 610.

### 2.3. SEM Characterization

Scanning electron microscopy (SEM) was used to assess the morphological characterization of the films. The experiments were performed using a Leo 1530 VP SEM (Oberkochen, Germany). All samples were sputter-coated with gold for SEM imaging. All figures were obtained with an electron high tension (EHT) at 3.00 kV.

### 2.4. Fourier-Transform Infrared (FTIR)Spectrometry 

A Bruker Tensor 27 Fourier-Transform Infrared (FTIR) Spectrometer (Billerica, MA, USA) was used, equipped with a deuterated triglycine sulfate detector and a multiple reflection, horizontal MIRacle ATR attachment (using a Ge crystal, from Pike Tech. (Madison, WI, USA)) that was continuously purged with nitrogen gas. Readings were taken at a range of 4000 to 400 cm^−1^ with 64 background scans and 64 sample scans at a resolution of 4 cm^−1^. For each film sample, four total measurements were taken to ensure homogeneity. However, only one spectrum is shown in this report to demonstrate the overall trend. Between samples, the ATR crystal was cleaned with methanol. 

### 2.5. Differential Scanning Calorimetry (DSC)

Data were collected using a TA Instruments (New Castle, DE, USA) Q100 DSC, with purged dry nitrogen gas flow (50 mL/min), equipped with a refrigerated cooling system. The instrument had been previously calibrated with indium for heat flow and temperature, and aluminum and sapphire reference standards were used to calibrate heat capacity. After washing, film samples were cut and encapsulated in aluminum pans and heated in the DSC. Temperature-modulated differential scanning calorimetry (TMDSC) measurements were taken at a heating rate of 2 °C/min with a modulation period of 60 s and temperature amplitude of 0.318 °C, from −40 to 400 °C. Test specimens of 5–6 mg were cut into several pieces with diameters ~1 mm and sampled to include a mixture illustrative of the bulk film composition to mitigate potential divergent data.

### 2.6. Thermogravimetric Analysis (TGA)

The degradation of films was monitored using a Pyris 1 Thermogravimetric Analyzer (PerkinElmer, Waltham, MA, USA) with a nitrogen gas flow rate of 50 mL/min. Changes in mass were recorded over a temperature range of 25 to 500 °C at a rate of 5 °C/min. Test specimens of 8–10 mg were cut into several pieces with diameters ~1 mm and sampled to include a mixture illustrative of the bulk film composition to mitigate divergent data.

## 3. Results and Discussion

### 3.1. Surface Morphology

The composite silk films produced by nylon 610 (LNS) greatly resembled those of nylon 1010 (HNS) blends at every composition ratio. Both types of nylon homopolymer produced chalk white film, whereas the silk homopolymer produced translucent tan film. Blended films adopted an appearance believed to be the average result of its constituents. 

Miscibility plays an important role in the overall structure and properties of polymer blends for designed application purposes [22]. Visual observation suggested adequate miscibility at all ratios except 50/50 blends in both nylons which might experience slight phase separation. Liu et al. [30] similarly reported silk and nylon 66 to be nearly miscible in all ratios except 1:1. Phase separation in LNS50 was experienced through what appeared to be silk fibroin aggregates suspended in a nylon network, whereas HNS50 was arranged in the opposite way. Both blends had a 50% silk composition.

**Figure 1:**
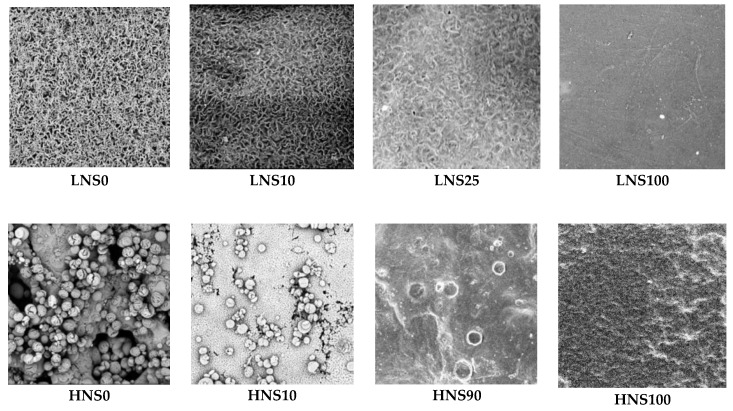
Selected scanning electron microscopy (SEM) images of (top) nylon610-silk (LNS) blend films and (bottom) nylon1010-silk (HNS) blend films. (each image scale is 150 µm ×150 µm).

SEM was performed to analyze the microstructure of the film surfaces. As seen in Figure 1, the microstructure assumed by nylon 610 (LNS) was dramatically different from that of nylon 1010 (HNS). LNS0 showed that pure nylon 610 produced fibril-like features, whereas HNS0 (pure nylon 1010) was found to be more particle-like at the micrometer scale. A closer examination of HNS0 revealed an almost sponge-like matrix. 

Generally, LNS blends produced more homogeneous films compared to HNS. This is likely due to differences in molecular chain length. It is possible that the lower molar mass of nylon 610 resulted in higher hydrogen bonding per unit mass. LNS100 and HNS100 were both made of silk homopolymer, however LNS100 was fabricated at 15% *w*/*v* as opposed to 10% *w*/*v* in HNS100. LNS100 shows a very smooth surface, whereas HNS100 is more wrinkled. The increased wrinkling can be explained by a greater mass loss due to evaporation of more solvent.

### 3.2. Structural Analysis

**Figure 2:**
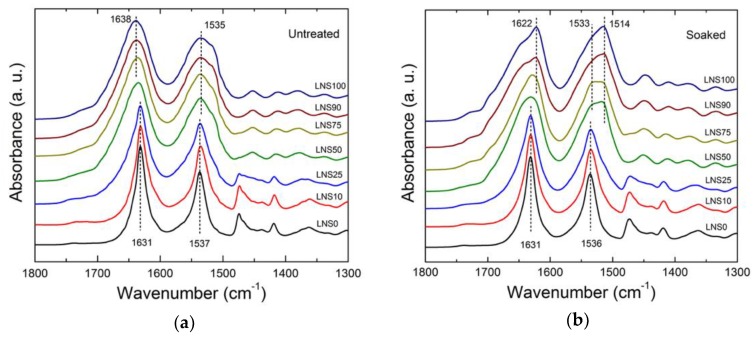
Fourier- transform infrared (FTIR) spectra of nylon610-silk (LNS) films (**a**) before and (**b**) after water bath treatment.

**Figure 3:**
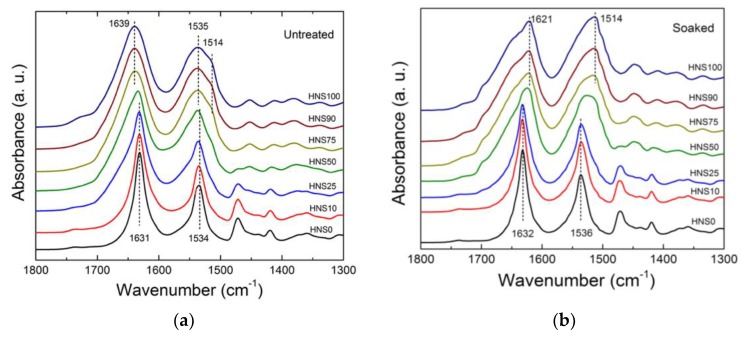
FTIR spectra of nylon1010-silk (HNS) films (**a**) before and (**b**) after water bath treatment.

To gain a better understanding of the interactions between the silk and nylon molecular chains as well as the effects of fabrication processing on structural confirmation, FTIR was used to examine the amide groups of silk fibroin and both nylon 610 and 1010 blends. Figure 2 and Figure 3 show the amide I and amide II regions of the IR spectra of the different silk/nylon composite films before and after the removal of CaCl_2_ using a 30 min water bath treatment.

Both nylon 610 (LNS0) and nylon 1010 (HNS0) showed an absorption band at 1631 cm^−1^, which is attributed to the characteristic alpha-crystalline conformation of nylons [32,33]. The location of this absorption band only slightly altered (from 1631 to 1632 cm^−1^) upon removal of CaCl_2_ in the water bath. SF showed an absorption band at 1639 cm^−1^ (amide I) prior to water wash, indicative of dominated random coils and extended chains in the protein structure [34]. Upon CaCl_2_ removal, however, the amide I peak shifted to 1622 cm^−1^ indicating that an intermolecular beta-sheet conformation was formed [20]. The shoulder observed at approximately 1655 cm^−1^ was attributed to C=O stretching in the SF. For unwashed SF and Nylon/SF blends, no significant peak could be observed at 1622 cm^−1^, suggesting that beta-sheet secondary structures were formed by removing CaCl_2_ in the water bath.

For nylon-dominated samples in the amide II region, the effect of removing CaCl_2_ is once again negligible (from 1537, 1534 to 1536 cm^−1^). On the other hand, in samples containing a greater silk composition, a band observed at 1535 cm^−1^ representing the amorphous phase was shifted to 1514 cm^−1^, which has been reported to represent water inaccessible beta-sheets and tyrosine side chain groups [35]. The observations discussed suggest a unique interaction between silk and nylon chains which is affected by the removal of CaCl_2_ molecules. 

### 3.3. Thermogravimetric Analysis

**Figure 4:**
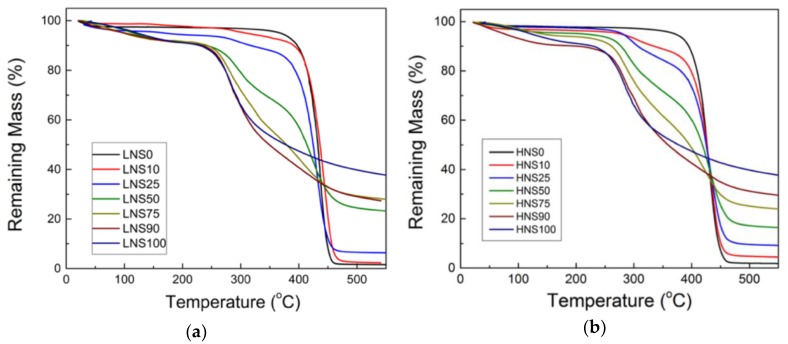
Thermogravimetric analysis demonstrates mass loss of (**a**) silk-nylon 610 (LNS) and (**b**) silk-nylon 1010 (HNS) blend films.

Strong interactions between polymers of composite blends can result in enhanced properties of the blends [29]. Thermogravimetric analysis was conducted on the solution cast films to investigate how their thermal stabilities were affected by the intermolecular interactions suggested by structural analysis. Figure 4 shows mass loss during heating as a function of temperature for silk, nylon 610, nylon 1010, and the various silk/nylon composites.

In both types of composite films, a multi-step mass loss mechanism is observed. The degradation behaviors of both blend types appear to follow a trend intuitive of their composition. In the silk fibroin homopolymer, there is an experienced mass loss beginning at approximately 265 °C, whereas LNS0 and HNS0 remain thermally stable until approximately 400 °C. Although silk begins to degrade at a lower temperature than the nylons, Figure 4 shows that the SF homopolymers retained 40% of its mass when heated up to 500 °C, whereas in both types of nylon virtually 0% mass remained at 500 °C. 

A visual interpretation of the 500 °C region in Figure 4 suggests that linear tunability can be achieved in HNS samples. The percent mass remaining values for the different blends suggest that the thermal contributions from each component are strictly additive (e.g., HNS30 mass at 500 °C = HNS100 (silk) mass × 30% + HNS0 (nylon 1010) mass × 70%, both at 500 °C). However, this was not observed to such extent in the LNS samples. Instead, the data were grouped together and unproportioned to their composition ratios. This observation may be indicative of some synergistic interaction in some of the LNS blends. To gauge the influence of the interaction, the measured values for percent mass remaining at 500 °C are compared against corresponding expected values in Table 1.

Expected values were determined under the assumption that no interactions were present. Therefore, any measured value that is not within reasonable deviation of the expected value should be considered as having synergism [29]. The expected percent mass remaining at a given temperature, *M_E_(T)* is determined from Equation (1):

(1) $$M_{E}\left(T\right)=M_{S}\left(T\right)\Phi_{S}+M_{N}\left(T\right)\left(1-\Phi_{S}\right),$$

where *M_S_(T)* and *M_N_(T)* are the percent mass remaining at temperature *T*, for silk protein and nylon respectively, and $\Phi_{S}$ is the percent composition of silk. With the exception of LNS50, the measured mass remaining at 500 °C is less than the expected value for each ratio. This indicates a decreased thermal stability in the materials and suggests some negative synergism arising from chain interactions. Clearly, LNS50 is the only sample with the measured mass remaining higher than expected. Since this sample experienced some macrophase separation (as mentioned in the morphology section), this could suggest that the sample tends to degrade slowly or heterogeneously if silk and nylon have less interactions. Of course, an overinterpretation of this result should be avoided, since the authors do not have a fully miscible 50/50 blend for comparison. Nevertheless, the considerable differences between measured and expected values support results from structural analysis suggesting a strong interaction between silk and the nylons, which can clearly affect their thermal degradation process. 

### 3.4. DSC Analysis

**Figure 5:**
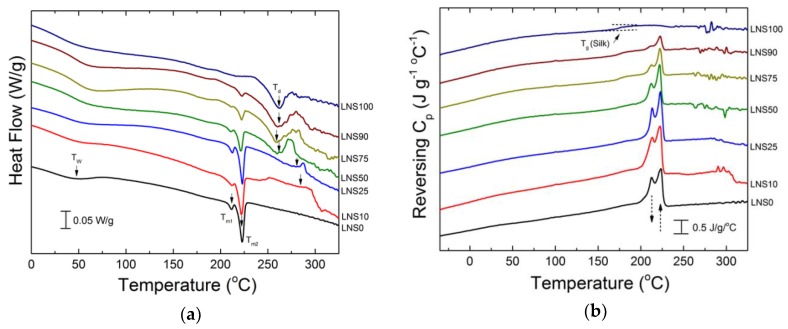

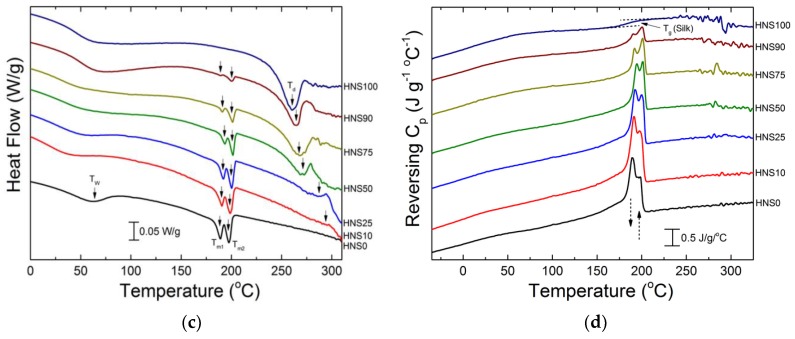
Differential scanning calorimetry (DSC) heat flow and reversing heat capacity of (**a**,**b**) LNS films and (**c**,**d**) HNS films.

Further analysis was performed using a temperature modulated DSC to investigate phase behavior in the films. Normalized heat flow vs. temperature results for the different films are shown in Figure 5 and have been scaled vertically for clarity. Shown in Figure 5a,c, the two visible exotherms between 170 and 230 °C indicate the presence of both metastable gamma-phase and stable alpha-phase crystals in both nylons. 

Relative peak intensities suggest that the nylon 610 homopolymer (LNS0) is alpha-phase dominated. In all films containing nylon 610, an initial melting of metastable γ crystal phase (*T*_m1_ peak) occurs at 213 °C, followed by a second melting of stable α crystals (*T*_m2_ peak) at 223 °C. No shift in the melting temperatures was observed at different composition ratios; however, with increasing concentrations of silk, there appeared to be a decrease in γ-phase crystals and an increase in α crystals.

In contrast to nylon 610, nylon 1010 (HNS0) was dominated by gamma-phase crystals. Similar to LNS films, HNS films experienced a decrease in gamma crystals and an increase in alpha-phase crystals, although the effects appeared more pronounced. Unlike LNS samples, HNS samples also exhibited variable melting temperatures when blended with different ratios of silk. Table 2 shows melting and crystallization temperatures for different HNS films. Increased temperatures were observed as composition ratios approached 50/50 from both directions. HNS0 had a *T*_m1_ and *T*_m2_ of 189.0 and 197.5 °C, respectively, compared to *T*_m1_ and *T*_m2_ of 189.1 and 200.1 °C, respectively, in the HNS90 sample. HNS50 displayed the highest melting temperatures out of the nylon 1010-silk films at *T*_m1_ = 193.5 and *T*_m2_ = 201.5 °C.

### 3.5. Mechanism

CaCl_2_–formic acid solvent has been used to produce regenerated SF materials with improved mechanical strength [36] and thermal properties [20]. However, the presence of CaCl_2_ has also been reported to decrease beta-sheet crystallinity in silk fibroin films and cause more random coils. Murase [37] explained this to be the result of Ca^+^ ions infiltrating the silk structure, thereby preventing hydrogen bonding. 

**Figure 6:**
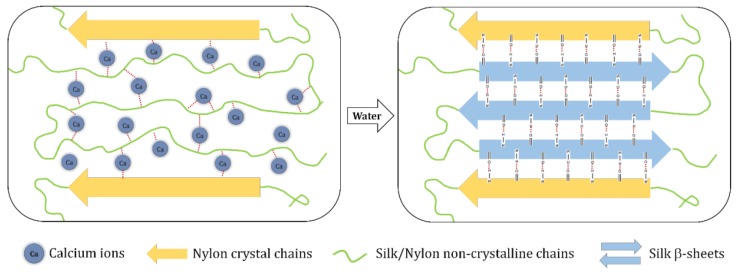
Proposed mechanism for silk-nylon molecular interactions induced by removing CaCl_2_ in water.

For this study, the authors prepared blend films of silk with nylons using a CaCl_2_–formic acid solution cast followed by water treatment. FTIR analysis showed that the removal of Ca^+^ ions induced beta-sheet formation in samples with significant silk composition. On the basis of experimental results and insight from previous investigations [20,36], the authors offer the following mechanism to illustrate beta-sheet formation in the silk films (see Figure 6). They propose that calcium chloride disrupts hydrogen bonding in silk and some of the nylon molecules and, once removed, it allows for hydrogen bonding between itself and nylon to create a blend film. They suggest that the initial presence of calcium chloride is crucial to first disrupt intramolecular bonding in silk so that intermolecular hydrogen bonding between silk and nylon is possible. 

## 4. Conclusions

In this study, the authors showed that nylon610-silk and nylon1010-silk blend films could be prepared by solution casting from CaCl_2_–formic acid. The removal of CaCl_2_ was observed to induce beta-sheet formation in the silk as indicated by the 1622 cm^−1^ peak in FTIR. Strong interactions between silk and nylon led to a tunable thermal stability in composites as shown by the TGA and DSC. Increasing the concentration of silk also led to a decrease in metastable gamma nylon crystals and an increase in stable alpha nylon crystals. This effect was especially pronounced in HNS films where heightened melting temperatures were also observed approaching 50/50 composition. It is possible that the addition of silk causes changes in the nylon side chains, further leading to the appearance of alpha crystals rather than gamma crystals. More studies will be conducted in the future to further understand this mechanism.

## Figures and Tables

**Table 1:** Thermogravimetric analysis (TGA) results showing measured vs. expected values for mass remaining at 500 °C.

Sample	Silk/%	Measured M_500 °C_/%	Expected M_500 °C_/%
LNS0	0%	1.7%	1.7%
LNS10	10%	2.6%	5.5%
LNS25	25%	6.6%	11.3%
LNS50	50%	24.5%	20.9%
LNS75	75%	29.4%	30.4%
LNS90	90%	29.0%	36.2%
LNS100	100%	40.0%	40.0%
HNS0	0%	2.0%	2.0%
HNS10	10%	4.8%	5.8%
HNS25	25%	9.6%	11.5%
HNS50	50%	17.2%	20.9%
HNS75	75%	25.1%	30.4%
HNS90	90%	31.2%	36.0%
HNS100	100%	39.8%	39.8%

**Table 2:** Melting and crystallization temperatures determined from DSC.

Sample	Silk Composition	*T*_c1_	*T*_c2_	*T*_m1_	*T*_m2_	*T*_d_ (°C)
HNS0	0%	189.5	197.3	189.0	197.5	-
HNS10	10%	191.4	198.4	190.6	198.8	290
HNS25	25%	192.4	199.4	192.0	200.3	287
HNS50	50%	194.5	200.8	193.5	201.5	272
HNS75	75%	192.1	200.7	191.1	201.2	270
HNS90	90%	190.3	200.5	189.1	200.1	269
HNS100	100%	-	-	-	-	267
